# Zero Suicide Model Implementation and Suicide Attempt Rates in Outpatient Mental Health Care

**DOI:** 10.1001/jamanetworkopen.2025.3721

**Published:** 2025-04-07

**Authors:** Brian K. Ahmedani, Robert B. Penfold, Cathrine Frank, Julie E. Richards, Christine Stewart, Jennifer M. Boggs, Karen J. Coleman, Stacy Sterling, Bobbi Jo H. Yarborough, Gregory Clarke, Michael Schoenbaum, Erika M. Aguirre-Miyamoto, Lee J. Barton, Hsueh-Han Yeh, Joslyn Westphal, Sarah McDonald, Arne Beck, Rinad S. Beidas, Laura Richardson, Jacqueline M. Ryan, Edward T. Buckingham, Stuart Buttlaire, Cambria Bruschke, Jean Flores, Gregory E. Simon

**Affiliations:** 1 Center for Health Policy & Health Services Research, Henry Ford Health, Detroit, Michigan; 2 Behavioral Health Services, Henry Ford Health, Detroit, Michigan; 3 Health Research Institute, Kaiser Permanente Washington, Seattle; 4 School of Public Health, University of Washington, Seattle; 5 Institute for Health Research, Kaiser Permanente Colorado, Castle Rock; 6 Kaiser Permanente Southern California, Pasadena; 7 Kaiser Permanente Bernard J. Tyson School of Medicine, Pasadena, California; 8 Kaiser Permanente Northern California, Oakland; 9 Department of Psychiatry and Behavioral Sciences, University of California, San Francisco; 10 Center for Health Research, Kaiser Permanente Northwest, Portland, Oregon; 11 National Institute of Mental Health, Bethesda, Maryland; 12 Department of Medical Social Sciences, Northwestern University Feinberg School of Medicine, Chicago, Illinois; 13 Kaiser Permanente, National, Oakland, California; 14 Johns Hopkins Bloomberg School of Public Health, Baltimore, Maryland

## Abstract

**Importance:**

Suicide is a major public health concern, and as most individuals have contact with health care practitioners before suicide, health systems are essential for suicide prevention. The Zero Suicide (ZS) model is the recommended approach for suicide prevention in health systems, but more evidence is needed to support its widespread adoption.

**Objective:**

To examine suicide attempt rates associated with implementation of the ZS model in outpatient mental health care within 6 US health systems.

**Design, Setting, and Participants:**

This quality improvement study with an interrupted time series design used data collected from January 2012 through December 2019, from patients aged 13 years or older who received mental health care at outpatient mental health specialty settings within 6 US health systems located in 5 states: California, Oregon, Washington, Colorado, and Michigan. Analyses were conducted from January through December 2024.

**Exposure:**

The ZS model was implemented in 4 health systems at different points during the observation period (2012-2019) and compared with health systems that implemented the model before the observation period (postimplementation). Implementation included suicide risk screening, assessment, brief intervention (safety plan, means safety protocol), and behavioral health treatment.

**Main Outcomes and Measures:**

The primary outcome was a measure of standardized monthly suicide attempt rates captured using health system records and government mortality records. Suicide death rates were also measured as a secondary outcome.

**Results:**

There was a median of 309 107 (range, 55 354-451 837) unique patients per month. In 2017, there were 317 939 eligible individuals (63.2% female). Baseline suicide attempt rates were at least 30 to 40 per 100 000 individuals at each implementation site and decreased to less than 30 per 100 000 individuals at 3 sites by 2019. Decreases in suicide attempt rates were observed at 3 intervention health systems after site-specific implementation: health systems A and B had decreases of 0.7 per 100 000 individuals per month and C, 0.1 per 100 000 individuals per month. System D evidenced a similar suicide attempt rate after implementation (before implementation: median rate: 35.0 [range, 11.0-50.3] per 100 000 patients per month; after implementation: median rate: 34.3 [range, 18.5-42.0] per 100 000 patients per month). The 2 postimplementation health systems maintained low or declining suicide attempt rates throughout the observation period. The rate at system Y decreased by 0.3 per 100 000 individuals per month across the observation period. The rate at system Z began at 11 per 100 000 individuals per month and declined by 0.03 per 100 000 individuals per month during the observation period. Two systems evidenced reductions in the suicide death rate after implementation: system B declined by 0.2 per 100 000 individuals per month and system C by 0.1 per 100 000 individuals per month.

**Conclusions and Relevance:**

In this quality improvement study, ZS model implementation was associated with a reduction in suicide attempt rates among patients accessing outpatient mental health care at most study sites, which supports widespread efforts to implement the ZS model in these settings within US health systems.

## Key Points

**Question:**

Is implementation of the Zero Suicide model in outpatient mental health care associated with reductions in suicide attempts?

**Findings:**

This quality improvement study of 55 354 to 451 837 individuals per month aged 13 years or older found that implementation of the Zero Suicide model was associated with a reduction in suicide attempt rates in 3 of 4 health systems, while the fourth system experienced a lower sustained rate. Two systems that implemented the model before the observation period maintained low or declining rates.

**Meaning:**

Findings from this study support implementation of the Zero Suicide model in outpatient mental health care.

## Teaser

This quality improvement study evaluates whether implementing the Zero Suicide model in outpatient mental health care is associated with lower suicide attempt rates among individuals aged 13 years or older.

## Introduction

Suicide is a major public health concern in the US and across the world.^1^ Annual US suicide rates were 25% higher in 2021 compared with 2000.^2^ Large-scale approaches are urgently needed to address this challenge.

In 2024, the US Surgeon General released a new national strategy for suicide prevention.^3^ The report called on health systems to be active in suicide prevention; goal 8 is to “implement effective suicide prevention services as a core component of health care.” Data show that 83% of individuals have health care visits before suicide, and 92% make contact with the health system before a suicide attempt.^4,5^ The Zero Suicide (ZS) model has become the preferred suicide prevention approach in health systems.^6^ The first version of the ZS model was implemented in 2001.^7^ After implementation, the model was associated with a near 80% reduction in suicide deaths sustained for more than 10 years in behavioral health services.^7,8,9^ The full model involves a care pathway using a menu of evidence-based approaches. Patients are identified via suicide risk screening. Those identified as at risk receive a suicide risk assessment followed by a care pathway.^10,11,12,13,14,15,16,17^

The ZS model is referenced in national standards. The National Action Alliance for Suicide Prevention released a recommended minimum standard of care for suicide prevention corresponding to the ZS model.^18^ The Joint Commission released National Patient Safety Goal 15.01.01 requiring implementation of suicide risk screening and treatment for patients with behavioral health conditions and recommended it for all patients, citing elements of the ZS model.^19,20^ Variations of the ZS model have now been introduced in many US health systems.^21,22^ Previous research conducted at the health systems participating in the present study highlighted variations in implementation plans.^23^ This evidence showed that uptake was robust and occurred rapidly in outpatient mental health care and at various levels in other settings.^24^

Despite widespread adoption, little research has examined the relationship between ZS implementation and suicide outcomes, which is important in outpatient mental health, where most uptake has occurred. There is increasing urgency for evidence given rapid implementation momentum. Critics have raised concerns that the ZS model may be difficult to implement or create unrealistic expectations.^25,26,27,28^ However, advocates have pointed to evidence supporting ZS model interventions and that it has facilitated a unified movement.^6,29^ Data from Australia indicate that among individuals with prior suicide attempt, the ZS model was associated with reductions in re-attempts.^30,31^ There remains an urgent need for evidence on the association of ZS model implementation with suicide outcomes to inform health care adoption and policy.^23,32,33^ The present research aimed to fill these gaps in, to our knowledge, the largest study on ZS model implementation in outpatient mental health settings to date.

## Methods

### Population and Setting

Six health systems in California, Oregon, Washington, Colorado, and Michigan participated in the ZS model implementation study reported in this quality improvement study. The participating systems provided medical and behavioral health care to more than 10 million patients per year across clinical settings, including over 300 000 patients aged 13 years or older per month in outpatient mental health care. All systems were integrated care delivery and insurance systems with primary care and mental health care. The participating Kaiser Permanente systems in northern and southern California together served approximately 9 million patients, followed by Henry Ford Health in Michigan, serving 1.2 million, and Kaiser Permanente health care systems in Oregon, Washington, and Colorado, serving between 600 000 and 700 000 members each.^23,24^ These health systems have affiliated health plans providing commercial, public, and self-paid insurance and have access to electronic health record (EHR) and insurance claims data. The institutional review boards at the participating institutions approved data use with a waiver of documented informed consent because there was no participant contact and only existing data were used. The results reporting adhered to the Standards for Quality Improvement Reporting Excellence (SQUIRE) reporting guideline for quality improvement studies.

**Figure 1.**
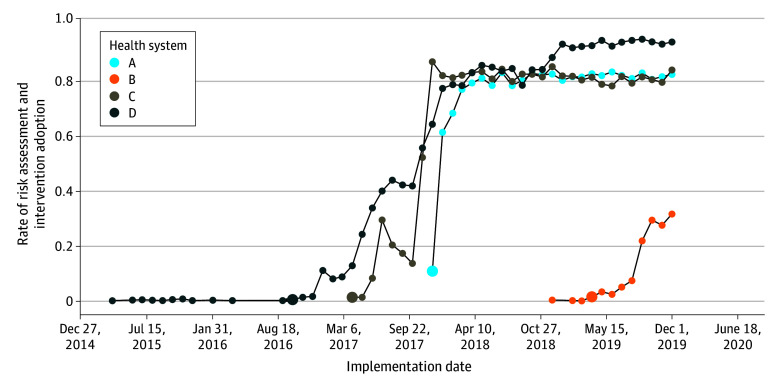
Time Point of Zero Suicide Model Implementation and Monthly Rates of Adoption of Risk Assessment in Outpatient Mental Health Care in 6 Participating Health Systems The implementation time point was defined as the month and year when health systems implemented a package of suicide risk assessment, safety planning, and care pathways for individuals who screened positive for suicide risk. The 4 large circles indicate the implementation start date at each site.

Embedded researchers within each system (B.K.A., J.M.B., K.J.C., S.S., B.J.H.Y., and G.E.S.) collaborated with care delivery stakeholders to document components of the ZS model implemented from January 2012 through December 2019.^23^ The participating health systems had unique implementation start dates and pace of adoption and prioritized different components. This study examined outcomes associated with implementation in outpatient mental health care at these systems. The 4 implementation group health systems implemented the ZS model during the observation period (2012-2019): system A in December 2017, system B in April 2019, system C in April 2017, and system D in October 2016. The 2 postimplementation group health systems implemented the ZS model prior to the observation period: system Y (Kaiser Permanente Washington) in 2012 and system Z (Henry Ford Health) in 2001. These time points were considered the go-live period of implementation in outpatient mental health care. As depicted in Figure 1, implementation was defined as the time point when each system began implementation of the care pathway for individuals with a positive suicide screening result in outpatient mental health settings. Rates of assessment adoption are the first step in the implementation package. The population for this study included individuals aged 13 years or older with an outpatient visit to a mental health specialty practitioner at the participating systems and who were members of the affiliated health plan for at least 10 of 12 months prior to the month of observation.

### The ZS Model

The ZS model is composed of a menu of evidence-based approaches along a care pathway starting with suicide risk screening and assessment followed by brief interventions and treatment. All participating systems chose to use the ninth item of the Patient Health Questionnaire–9 as the primary suicide risk screening question.^10^ The Columbia Suicide Severity Rating Scale was chosen as the assessment tool at 5 health systems,^11^ while Henry Ford Health used a local risk assessment composed of evidence-based factors. All systems implemented means safety protocols and safety planning interventions,^14,34^ and all provided outpatient mental health care, including evidence-based psychotherapy.^23,24^

### Design

A quality improvement study with an interrupted times series design was used to examine ZS model implementation.^35,36^ Figure 1 shows the timing of the implementation. Implementation was associated with moderate-high levels of fidelity to screening, clinical assessment, and brief intervention in outpatient mental health settings at the intervention systems (systems A-D).^24^ Among 4 674 515 eligible mental health visits in 2019, 3 416 904 visits (73%) included a suicide risk screening. Of 128 996 individuals who screened positive for suicide risk, 77% (99 098) received a risk assessment. Of 19 528 individuals with a positive risk assessment result, 82% (16 094) received a safety plan and/or means counseling.^24^

### Data Sources

Data were captured from the virtual data warehouse at each system.^37^ The virtual data warehouse is a federated data model combining EHR and insurance claims data using the same definitions and variables across sites. It consists of data on diagnoses, procedures, visit types, and administrative data recorded during clinical visits. Patient-reported demographics, including age, race and ethnicity, and sex were also captured. Race and ethnicity were defined as a combined variable captured during clinical visits. The race and ethnicity categories captured during clinical visits were African American or Black, American Indian or Alaska Native, Asian, Hawaiian or Pacific Islander, Hispanic, or White, other (optional category), and unknown (not recorded). Categories for sex were female, male, other (optional category), and unknown (not recorded). For each patient, census tract data were used to create geocoded variables on estimated income (low income if individuals resided in a census tract where the median household income was<$40 000) and estimated educational level (lower educational level if <25% of individuals in the tract had a college degree).

### Primary Outcome

The study included unique patients with an outpatient visit to a mental health specialty practitioner by month of the observation period. The primary outcome measure was a combined metric for suicide attempt and suicide death occurring within 90 days of that month. Suicide deaths (ascertained from government mortality records) included all deaths coded as intentional self-harm (*International Statistical Classification of Diseases and Related Health Problems, Tenth Revision* [*ICD-10*] codes X60-X84; Y87.0). Suicide attempts (ascertained from health system EHR and claims data) included any encounter with a diagnosis of intentional self-harm. Diagnosis data were captured using *International Classification of Diseases, Ninth Revision, Clinical Modification* codes from January 2012 through September 2015 and *International Statistical Classification of Diseases, Tenth Revision, Clinical Modification* codes from October 2015 through December 2019,^38^ corresponding to the *International Classification of Diseases, Ninth Revision* (*ICD-9*) to *ICD-10* transition in the US.^39^ Since suicide death is by definition considered a suicide attempt, a single monthly composite suicide attempt rate including all fatal and diagnosed nonfatal intentional self-harm events was created for analysis. As a secondary outcome, quarterly suicide death rates were also calculated separately. A 90-day measurement period is commonly used in other suicide prevention research and quality measurement and represents a clinically relevant time frame.

### Statistical Analysis

We calculated crude and standardized monthly suicide attempt rates per 100 000 patients with any outpatient mental health visit. Monthly suicide attempt rates were calculated for each health system. Patients with an outpatient mental health visit in each month were included in the denominator of that month. The numerator included patients in the denominator population for the month who experienced a suicide attempt within a period up to 90 days following that month. Direct standardization^40^ by age, sex, and race and ethnicity was used, with July 2017 serving as the standard population.

We used an interrupted time series design and segmented regression analyses^35^ to estimate the change in suicide attempt rates in the period before and after implementation of the ZS model. A secondary analysis using the same modeling approach was also conducted to evaluate change in quarterly suicide death rates.

The interruptions specified for system-specific models were derived from the observed rates of risk assessment and intervention delivery (Figure 1). We selected the first nonzero monthly rate of risk assessment to define the interruption, such that all individuals in the population were included in the denominator whether or not they received the full intervention. Separate models were fit for each system and interruption. Trend lines were also calculated for systems in the postimplementation group.

Segmented regression models controlled for baseline (preperiod) rates and trends in suicide attempts. We used maximum likelihood estimation to fit models and controlled for autocorrelation by including all significant autocorrelation parameters up to 12 months. Statistical model results are presented with the autocorrelation parameters assumed to be given. Backward elimination was used to include covariates in the final model, with a type 1 error threshold for inclusion of .05. A difference-in-differences approach was considered but could not be used as it violated the parallel trends assumption.^41,42^ All statistical analyses were performed from January through December 2024 using PROC AUTOREG in SAS, version 9.4 (SAS Institute Inc).^43^ Two-sided *P* ≤ .05 was considered statistically significant.

## Results

There was a median of 309 107 (range, 55 354 to 451 837) unique patients per month. As shown in Table 1, a total of 317 939 eligible individuals received outpatient specialty mental health care across 6 health systems in July 2017. The self-reported race and ethnicity composition of this population was 0.7% American Indian or Alaska Native (n = 2061), 7.1% Asian (n = 22 539), 8.0% Black or African American (n = 25 556), 0.6% Hawaiian or Pacific Islander (n = 1818), 22.8% Hispanic (n = 72 440), 58.2% White (n = 184 938), and 0.1% other (n = 418). Also, race and ethnicity were unknown for 2.6% of the population (n = 8169). This population was 63.2% female (n = 201 080), 36.7% male (n = 116 787), and less than 0.1% other (n = 50); 77.7% were 18 to 64 years of age (n = 247 145). Overall, 69.9% of the population (n = 222 151) had commercial insurance, 95.8% (n = 304 783) lived in neighborhoods with a median income above $40 000, and 67.8% (n = 217 600) lived in neighborhoods where less than 25% of residents had at least a 4-year college degree.

**Table 1.** Demographics of Patients With an Outpatient Mental Health Visit Across 6 Health Systems in July 2017^a^

Characteristic	Patients, No. (%) (N = 317 939)
Age group, y	
13-17	32 626 (10.3)
18-64	247 145 (77.7)
≥65	38178 (12.0)
Sex	
Female	201 080 (63.2)
Male	116 787 (36.7)
Other^b^	50 (<0.1)
Unknown	22 (<0.1)
Race and ethnicity	
American Indian or Alaska Native	2061 (0.7)
Asian	22 539 (7.1)
Black or African American	25 556 (8.0)
Hawaiian or Pacific Islander	1818 (0.6)
Hispanic	72 440 (22.8)
White	184 938 (58.2)
Other^b^	418 (0.1)
Unknown	8169 (2.6)
Insurance type	
Commercial	222 151 (66.9)
Medicaid	33973 (10.7)
Medicare	50 318 (15.8)
Private payer	10 110 (3.2)
Other	1387 (0.4)
Lower neighborhood income	13 156 (4.2)
Lower neighborhood educational level	100 339 (32.2)

^a^ Race and ethnicity were defined by patient self-report captured during health system visits. Income and education were derived from geocoded location based on census data. These data were used for direct standardization of the outcome in the main analyses.

^b^ Other was an optional category.

**Figure 2.**
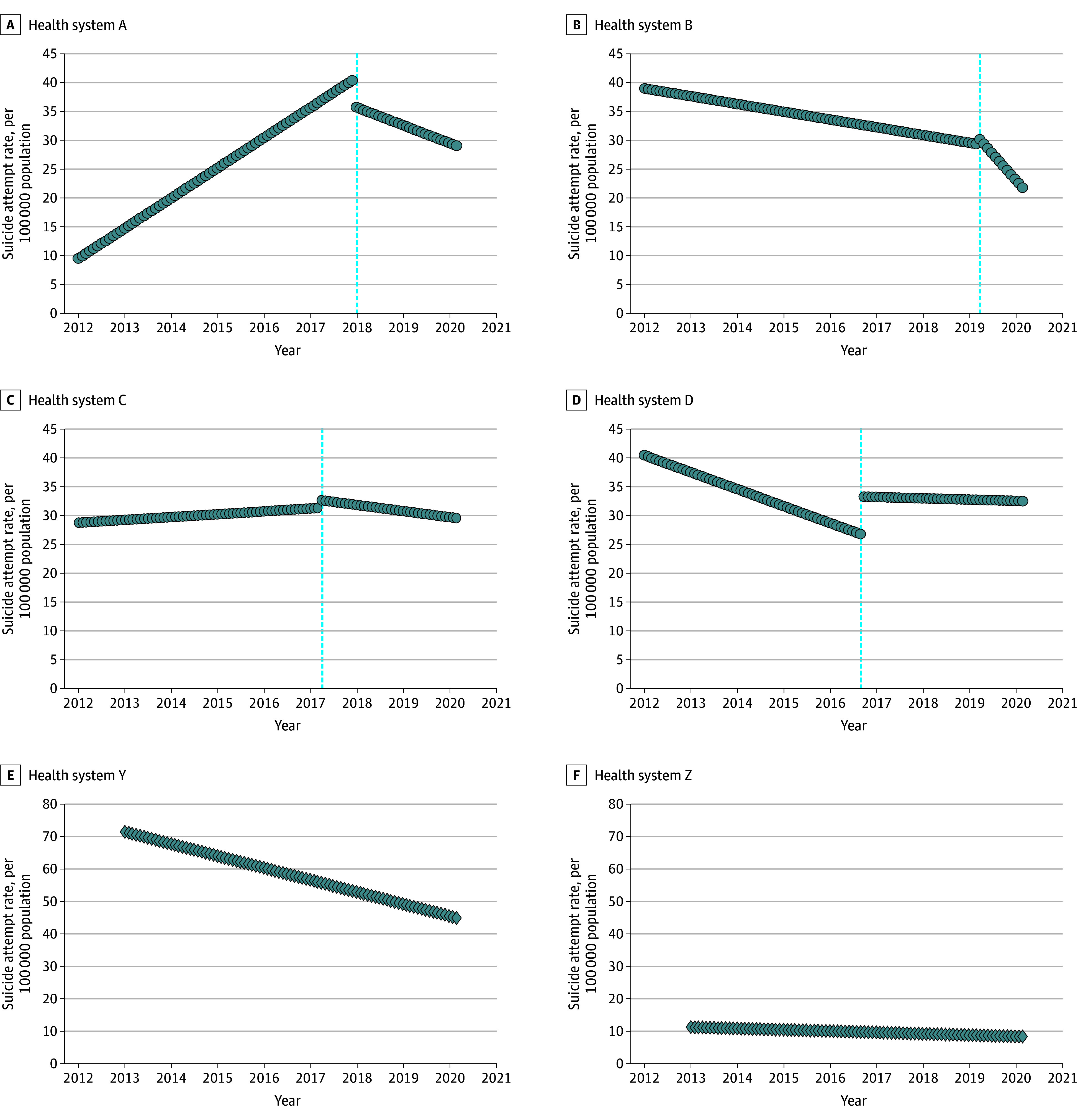
Interrupted Time Series Models for Health System–Specific Changes in Rates of Suicide Attempt Associated With Zero Suicide Model Implementation Composite suicide attempt rates for January in each year were calculated for patients receiving outpatient mental health care across 6 health systems. A-D, Health systems A through D implemented the Zero Suicide model in outpatient mental health care during the observation period between 2012 and 2019. Vertical dotted lines represent the month of Zero Suicide model implementation. E and F, Systems Y (Kaiser Permanente Washington) and Z (Henry Ford Health) implemented the Zero Suicide model in outpatient mental health care prior to the observation period.

Table 2 shows the results of statistical models measuring change in rates of suicide attempt following implementation of the ZS model. Baseline suicide attempt rates were at least 30 to 40 per 100 000 patients at each implementation site and decreased to less than 30 per 100 000 patients at 3 sites by 2019. Health systems A, B, and C had statistically significant reductions in suicide attempt rates following implementation (A and B: 0.7 per 100 000 patients per month; C: 0.1 per 100 000 patients per month). System B evidenced the largest decrease in suicide attempt rates by 0.7 per 100 000 patients per month. While system D did not show a reduction after implementation (before implementation: median rate; 35.0 [range, 11.0-50.3] per 100 000 patients per month; after implementation: median rate: 34.3 [range, 18.5-42.0] per 100 000 patients per month; *P* = .35), it did evidence a sustained lower plateaued suicide attempt rate after implementing the ZS model. Among the postimplementation group sites, system Y implemented the ZS model in 2012 (at the beginning of the observation window) and system Z implemented the model in 2001. System Y evidenced a declining rate of suicide attempts throughout the observation period (eg, from 71.8 per 100 000 patients in 2012 to 42.7 per 100 000 patients in 2020). System Z maintained the lowest sustained rate among all systems throughout the observation period, starting at 11.3 per 100 000 patients per month and declining to 0.3 per 100 000 patients per month. Suicide attempt rates are shown in Figure 2, with trend lines before and after ZS model implementation and marked intercepts at health system–specific implementation time points. Detailed versions of Figure 2 and a table of observed numbers of the outcome are available in the eFigure and eTable in Supplement 1.

**Table 2.** Interrupted Time Series Models Examining Suicide Attempt Rates During or After Zero Suicide Model Implementation^a^

Variable	Implementation health system												Postimplementation health system					
	A			B			C			D			Y			Z		
	Estimate (SE)	*t* Value	*P*r>|*t*|^b^	Estimate (SE)	*t* Value	*P*r>|*t*|^b^	Estimate (SE)	*t* value	*P*r>|*t*|^b^	Estimate (SE)	*t* value	*P*r>|*t*|^b^	Estimate (SE)	*t* value	*P*r>|*t*|^b^	Estimate (SE)	*t* value	*P*r>|*t*|^b^
Intercept	8.86 (3.51)	2.53	.03	39.10 (1.04)	37.70	<.001	28.73 (0.60)	48.32	<.001	40.72 (4.25)	9.58	<.001	71.80 (5.90)	12.09	<.001	11.30 (1.20)	9.30	<.001
Implementation (intercept)	−4.38 (5.55)	−0.79	.43	1.55 (2.55)	0.61	.55	1.45 (1.06)	1.36	.18	6.51 (6.03)	1.08	.29	NA	NA	NA	NA	NA	NA
Time	0.44 (0.08)	5.34	<.001	−0.11 (0.02)	−5.50	<.001	0.04 (0.02)	2.50	.01	−0.24 (0.13)	−1.94	.06	−0.30 (0.10)	−2.68	.01	−0.03 (0.01)	−1.40	.17
Time after implementation (slope)	−0.69 (0.33)	−2.12	.04	−0.65 (0.33)	−1.94	.05	−0.13 (0.04)	−3.03	.003	0.23 (0.24)	0.94	.35	NA	NA	NA	NA	NA	NA

Abbreviation: NA, not applicable.

^a^ Autoregression parameters were assumed to be given. Health systems A through D implemented the Zero Suicide model in outpatient mental health care during the observation period between 2012 and 2019: system A in December 2017, system B in April 2019, System C in April 2017, and system D in October 2016. These months represent the interruption time point for the segmented regression models in the interrupted time series analysis at each site. The postimplementation health systems implemented the Zero Suicide model in outpatient mental health care prior to the observation period: system Y (Kaiser Permanente Washington) in 2012 and system Z (Henry Ford Health) in 2001.

^b^ Probability of observing a value different from *t.*

Segmented regression models evaluating change in the secondary outcome of suicide death rates before and after ZS implementation are presented in Table 3. Health systems B and C demonstrated statistically significant reductions in suicide death rates. Health systems A and D did not show reductions in suicide death rate.

**Table 3.** Interrupted Time Series Models Examining Suicide Death Rates During Zero Suicide Model Implementation

Variable	Health system A			Health system B			Health system C			Health system D		
	Estimate (SE)	*t* Value	*P*r>|*t*|^a^	Estimate (SE)	*t* Value	*P*r>|*t*|^a^	Estimate (SE)	*t* Value	*P*r>|*t*|^a^	Estimate (SE)	*t* Value	*P*r>|*t*|^a^
Intercept	2.39 (0.29)	8.13	<.001	2.29 (0.17)	13.39	<.001	1.68 (0.22)	7.71	<.001	1.92 (0.57)	3.39	.002
Implementation (intercept)	−0.05 (0.02)	−3.03	.004	−0.02 (0.01)	−2.64	.01	<0.01 (0.01)	0.20	.84	−0.02 (0.04)	−0.51	.61
Time	1.47 (0.64)	2.31	.03	0.95 (0.42)	2.26	.03	0.31 (0.35)	0.89	.38	0.71 (0.84)	0.85	.40
Time after implementation (slope)	−0.08 (0.06)	−1.33	.19	−0.20 (0.06)	−3.69	.001	−0.08 (0.03)	−2.73	.009	−0.05 (0.06)	−0.74	.46

^a^ Probability of observing a value different from *t.*

## Discussion

This study provides, to our knowledge, the first multisite outcome evaluation of the ZS model in outpatient mental health settings. The findings indicated statistically significant reductions in suicide attempt rates after implementation of the ZS model across 3 of 4 large health systems. While the fourth system did not show a rate reduction, it did demonstrate a sustained lower suicide attempt rate after implementation. The 2 postimplementation health systems maintained low or continuously declining rates throughout the observation period. These findings are particularly important as the US embarks on implementing the 2024 National Strategy for Suicide Prevention and corresponding Federal Action Plan.^3^

These data support systematic implementation of the ZS model in outpatient mental health settings. Based on our previous work describing the components of the ZS model that were implemented in each health system,^23,24^ a ZS model care pathway should include systematic suicide risk screening at each outpatient mental health visit, followed by suicide risk assessment with a valid tool, safety planning and means reduction protocols, and access to evidence-based suicide prevention treatment. Since this study was implemented at the system level, it was not possible to evaluate the effect of each individual approach or intervention within the pathway. Nonetheless, there is already existing evidence to support each of these individual approaches.^10,11,14,16,17^ Consistent with national recommendations, implementation of the full care pathway within outpatient mental health settings is recommended for effective suicide prevention and treatment.^6^

As many health systems have begun implementing the ZS model, studies have documented a wide range of opportunities and barriers to implementation. Previous work demonstrated increases in screening and strong fidelity to the model workflow in outpatient mental health care.^24^ It is possible that if our health systems had even stronger or more rapid fidelity, they may have reached more individuals, leading to even greater reductions in suicide attempts. This may have uniquely impacted site D, which experienced a longer implementation ramp-up period compared with the other sites. A recent study of mental health clinicians in Australia found that practitioners thought the ZS model was a good approach to minimizing risk but acknowledged that successful implementation required adequate training and staffing resources as well as a strong safety culture.^26^

Another prior study found that health systems may have to consider adaptation of the ZS model to account for local or community circumstances.^32^ This process may require local teams choosing the best tools to fit their environment. This requirement aligns with the growing body of evidence that supports the importance of multimodal and health system–wide initiatives to prevent suicide.^44^ Future studies should examine the effects of these adaptations.

As health care settings continue to implement the ZS model, it will be essential to implement adequate quality measures to facilitate rapid quality improvement.^44^ There are unique challenges with measurement of variables necessary to evaluate implementation. While standard EHR systems are able to more easily measure screening and assessment, it is more difficult to assess the modality of psychotherapy or the quality of interventions delivered.^45^ Furthermore, there remain challenges in accessing timely mortality data, and these files are often costly to obtain.

While most research on ZS model implementation to date, including the present study, has been in outpatient mental health settings,^46^ future research is needed to examine implementation in other care settings. Data suggest that only 50% of people have a mental health diagnosis before suicide, and many health care visits prior to suicide occur in primary care.^5^ A recent study in Australia found that, among older adults, ZS model approaches may be needed in primary care settings, where much of mental health care occurs.^47^ As adaptations of the core ZS model are developed and implemented, special attention is needed to ensure that implementation is equitable across diverse communities.^48^

### Limitations

This study must be considered in the context of limitations. First, the study was conducted within 6 large health systems with insured populations. Findings may not directly reflect efforts in other types of health settings. Second, the data were limited to system-level outcomes. Individual-level results could not be assessed. Third, the study was conducted during the transition from *ICD-9* to *ICD-10* coding, with changes in coding practices potentially affecting the results. While some suicide attempts could have been miscoded across the entire study period, research indicates that suicide attempts were more likely to be undercounted in the *ICD-9* era, which coincides with a large portion of the preimplementation period in this study^38,39^ This result suggests that findings in this study may be conservative and that the actual effect estimates may have been greater than observed given that rates during the preimplementation phase may have been disproportionately higher than measured. Furthermore, in the *ICD-10* mortality coding scheme, some suicide deaths could also have been miscoded. Fourth, this study did not apply a randomized design, which could introduce the possibility of unmeasured biases. This was a clinical practice evaluation in which each health system determined when and how to implement the ZS model. The evaluation allowed examination of the change in trend and intercept comparing suicide attempt rates before and after implementation. Fifth, the study used a 90-day suicide attempt outcome. An alternate observation period may have produced different results. Sixth, despite the large sample size, power was limited to detect a difference in the secondary outcome of suicide death rate at systems A and D. Seventh, additional emphasis on suicide prevention after launch may have improved suicide attempt coding after implementation, such that suicide attempts prior to launch may be disproportionately undercoded. This may have led to more conservative results overall and particularly impacted system D, which experienced a plateaued suicide attempt rate after implementation.

## Conclusions

This quality improvement study, to our knowledge, was the largest evaluation of the ZS model implementation in US health systems. Implementation of the ZS model was associated with a reduction in suicide attempt rates among patients accessing outpatient mental health care at most study sites. Reductions in the suicide death rate were found at 2 sites after implementation. These findings reinforce the widespread implementation of the ZS model under way across the US and around the world.

## Notes

Supplement 1. **eFigure.** Suicide Attempt Rates Before and After Zero Suicide Model Implementation by Site **eTable.** Quarterly Numbers of Suicide Attempts Captured During the Observation Period at Zero Suicide Implementation Sites, 2012-2019

Supplement 2.
Data Sharing Statement
