# Supplementary material for: Zero Suicide Model Implementation and Suicide Attempt Rates in Outpatient Mental Health Care
Source: JAMA Netw Open. 2025 Apr 7;8(4):e253721. doi: 10.1001/jamanetworkopen.2025.3721 (PMC11976489; doi:10.1001/jamanetworkopen.2025.3721)
Supplement: Supplement 1. — eFigure. Suicide Attempt Rates Before and After Zero Suicide Model Implementation by Site eTable. Quarterly Numbers of Suicide Attempts Captured During the Observation Period at Zero Suicide Implementation Sites, 2012-2019 [file jamanetwopen-e253721-s001.pdf]

# Supplementary Online Content

Ahmedani BK, Penfold RB, Frank C, et al. Zero suicide model implementation and suicide attempt rates in outpatient mental health care. *JAMA Netw Open*. 2025;8(4):e253721. doi:10.1001/jamanetworkopen.2025.3721

eFigure. Suicide Attempt Rates Before and After Zero Suicide Model Implementation by Site

eTable. Quarterly Numbers of Suicide Attempts Captured During the Observation Period at Zero Suicide Implementation Sites, 2012-2019

This supplementary material has been provided by the authors to give readers additional information about their work.

© 2025 Ahmedani BK et al. *JAMA Network Open*.

**eFigure. Suicide Attempt Rates Before and After Zero Suicide Model Implementation by Site**

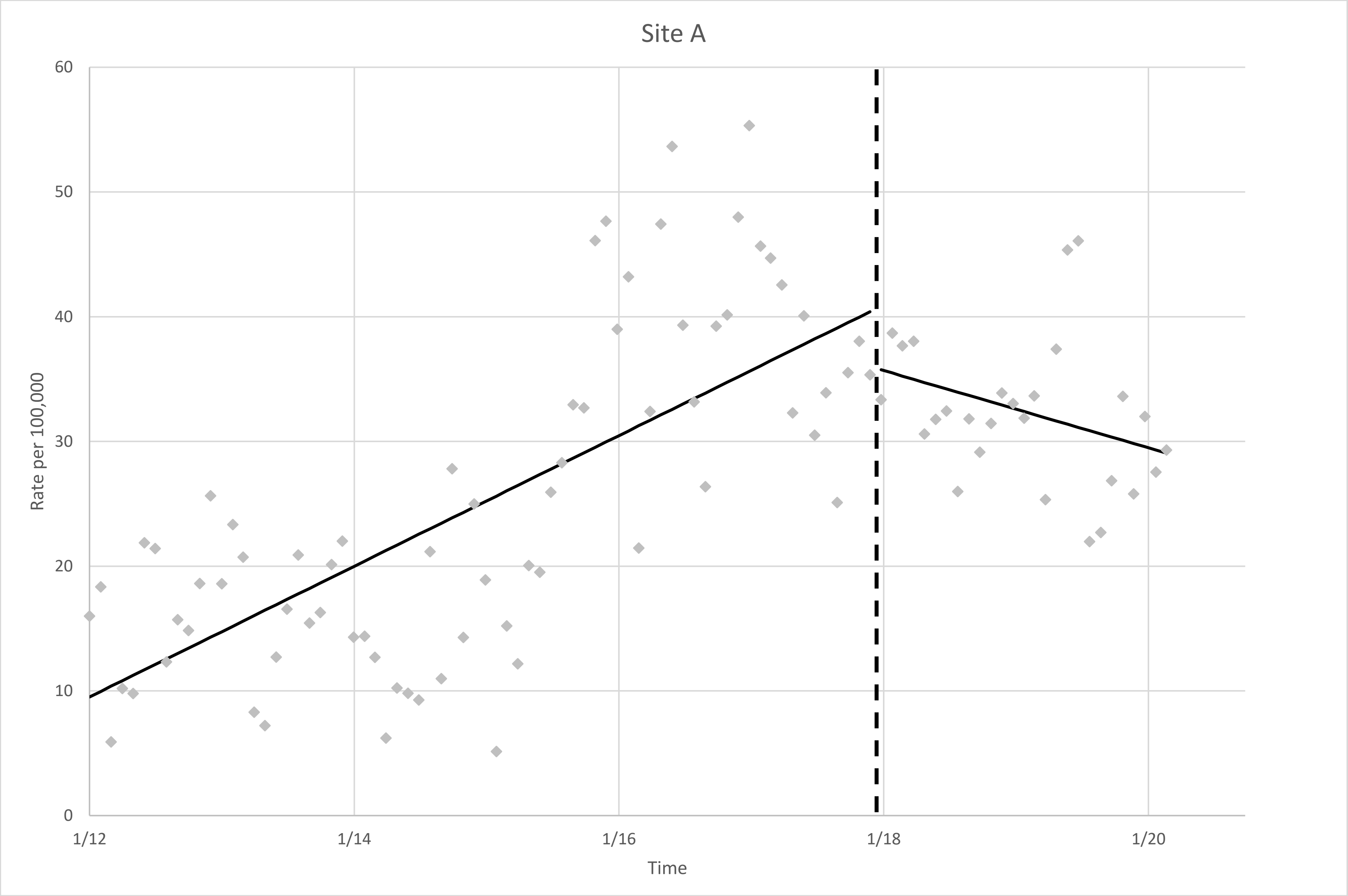


© 2025 Ahmedani BK et al. *JAMA Network Open*.

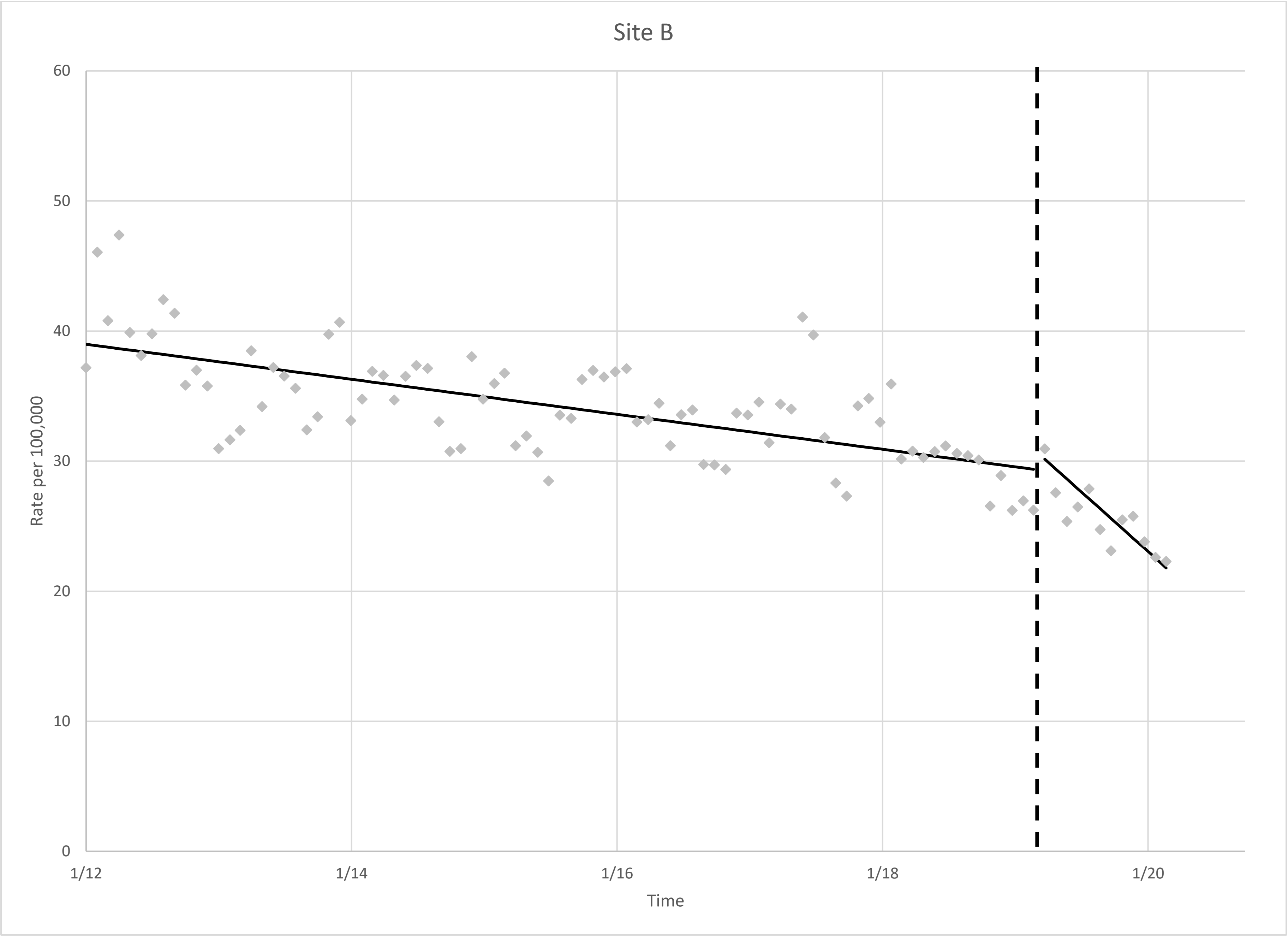


© 2025 Ahmedani BK et al. *JAMA Network Open*.

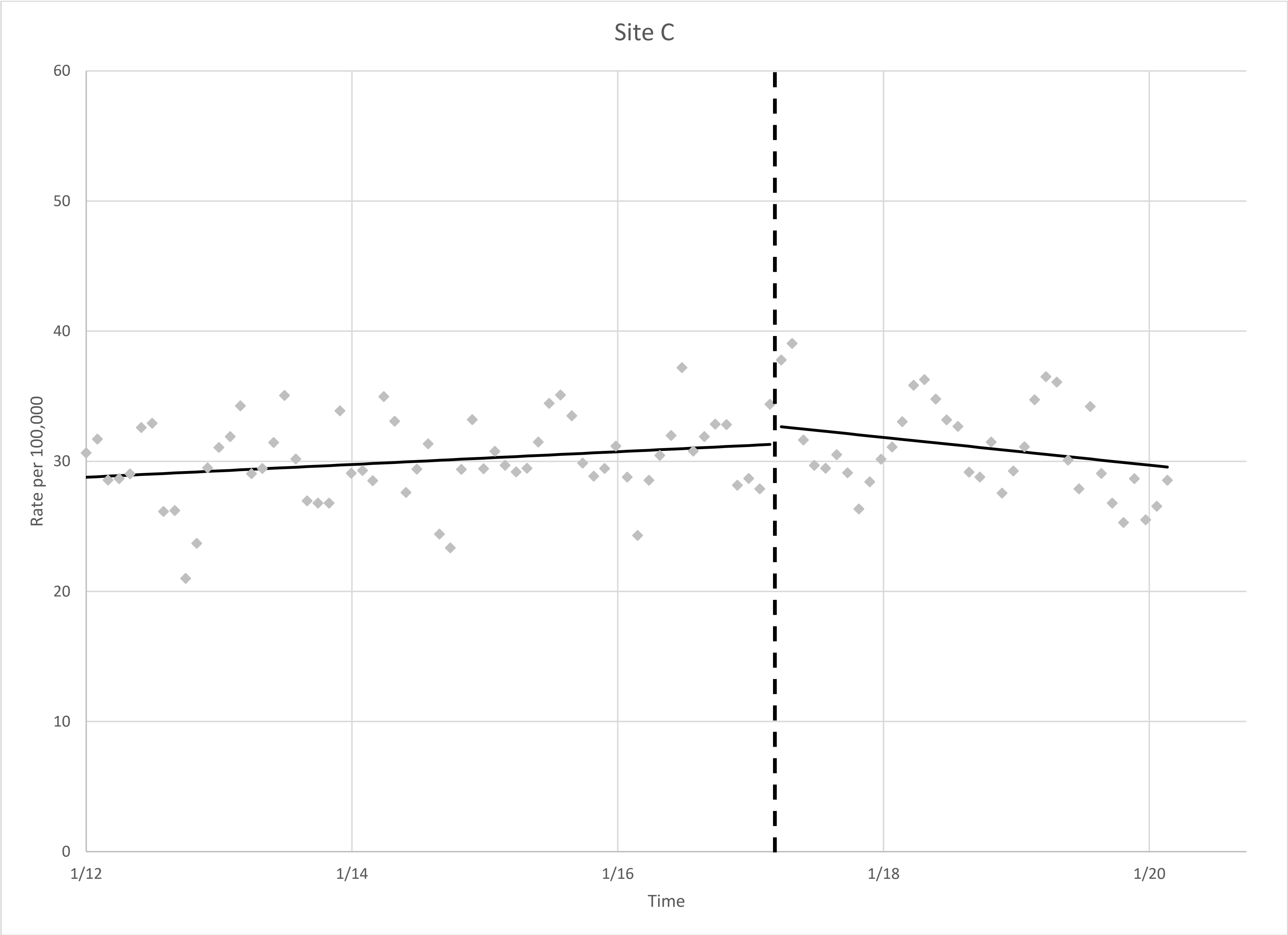


© 2025 Ahmedani BK et al. *JAMA Network Open*.

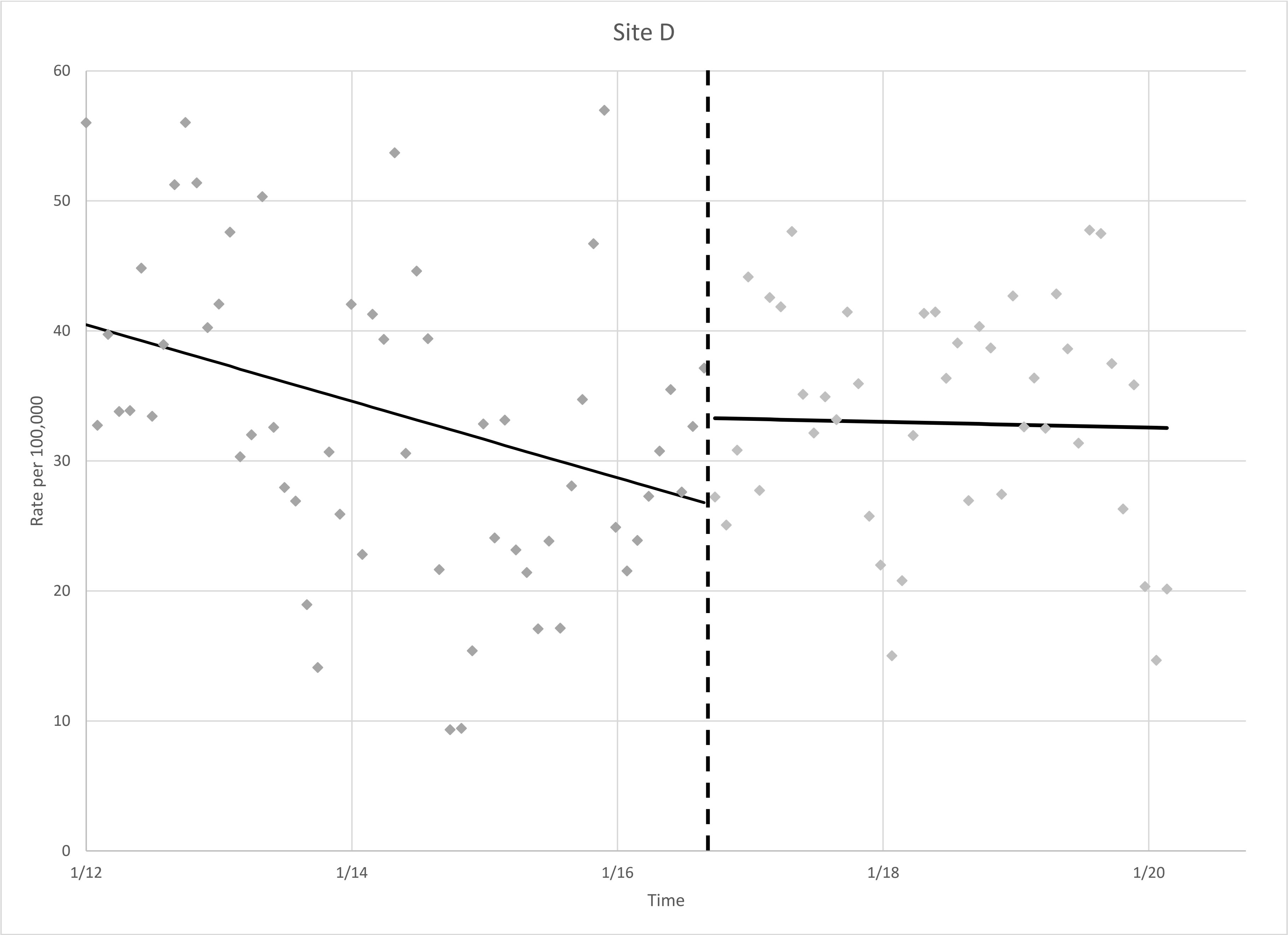


© 2025 Ahmedani BK et al. *JAMA Network Open*.

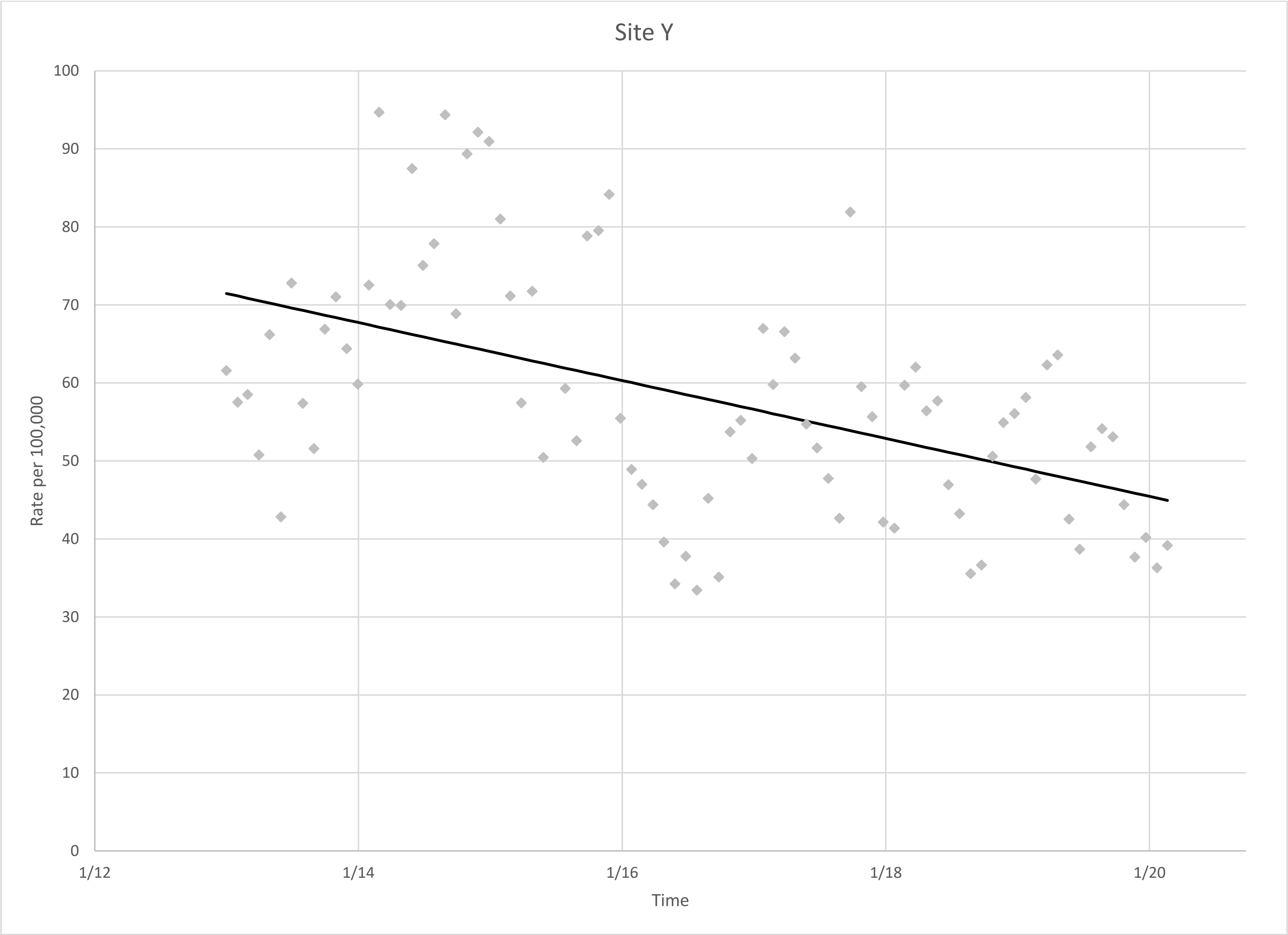


© 2025 Ahmedani BK et al. *JAMA Network Open*.

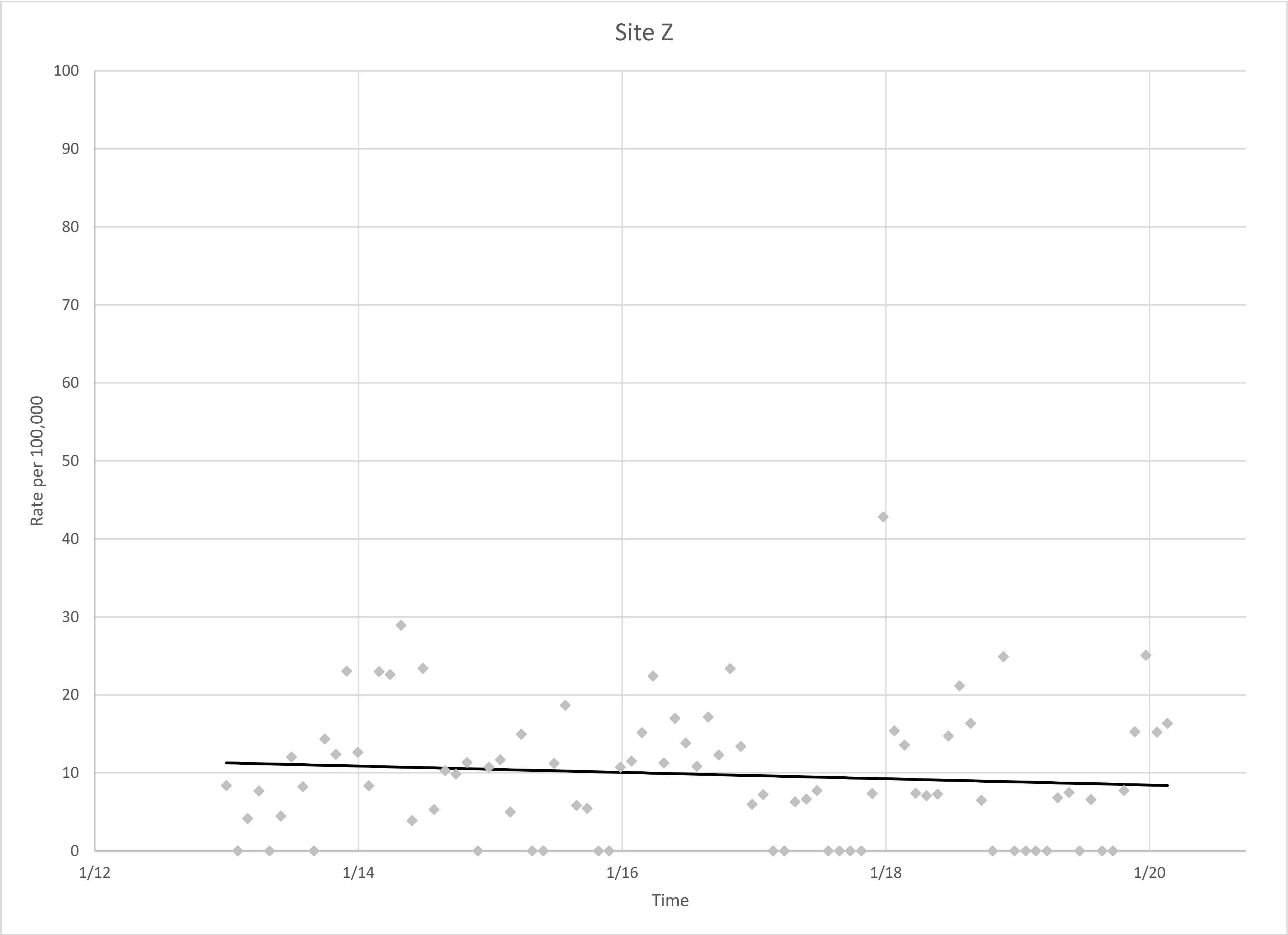


© 2025 Ahmedani BK et al. *JAMA Network Open*.

**eTable. Quarterly Numbers of Suicide Attempts Captured During the Observation Period at Zero Suicide Implementation Sites, 2012-2019**

| | Health System A | | Health System B | | Health System C | | Health System D | |
|---|---|---|---|---|---|---|---|---|
| Quarter | Definite self-harm or Suicide Death | Population with a mental health visit | Definite self-harm or Suicide Death | Population with a mental health visit | Definite self-harm or Suicide Death | Population with a mental health visit | Definite self-harm or Suicide Death | Population with a mental health visit |
| Jan-12 | 70 | 51,893 | 1,260 | 302,523 | 734 | 242,373 | 99 | 22,981 |
| Apr-12 | 70 | 50,499 | 1,278 | 305,883 | 726 | 241,375 | 86 | 23,019 |
| Jul-12 | 78 | 47,566 | 1,218 | 295,487 | 682 | 240,270 | 91 | 22,263 |
| Oct-12 | 94 | 48,735 | 1,044 | 288,372 | 578 | 237,586 | 120 | 23,843 |
| Jan-13 | 103 | 49,478 | 954 | 301,461 | 810 | 249,898 | 92 | 23,138 |
| Apr-13 | 49 | 52,652 | 1,144 | 312,381 | 771 | 257,560 | 95 | 24,647 |
| Jul-13 | 93 | 52,673 | 1,048 | 300,084 | 790 | 256,396 | 62 | 25,045 |
| Oct-13 | 98 | 50,899 | 1,087 | 288,620 | 720 | 249,051 | 61 | 26,491 |
| Jan-14 | 71 | 51,513 | 1,099 | 313,556 | 765 | 264,130 | 97 | 27,245 |
| Apr-14 | 47 | 54,187 | 1,173 | 326,047 | 851 | 266,394 | 113 | 27,415 |
| Jul-14 | 67 | 49,234 | 1,181 | 329,512 | 760 | 267,772 | 98 | 27,997 |
| Oct-14 | 108 | 47,240 | 1,040 | 314,052 | 722 | 254,818 | 34 | 30,864 |
| Jan-15 | 63 | 47,886 | 1,156 | 321,969 | 789 | 263,173 | 84 | 27,691 |
| Apr-15 | 84 | 49,138 | 1,079 | 345,235 | 850 | 282,587 | 61 | 29,650 |
| Jul-15 | 144 | 49,491 | 1,101 | 347,064 | 983 | 286,144 | 69 | 29,952 |
| Oct-15 | 226 | 53,748 | 1,209 | 330,687 | 817 | 277,774 | 133 | 29,308 |
| Jan-16 | 201 | 59,279 | 1,376 | 387,368 | 852 | 305,375 | 70 | 29,853 |
| Apr-16 | 271 | 60,808 | 1,342 | 407,563 | 938 | 309,031 | 92 | 29,595 |
| Jul-16 | 198 | 60,832 | 1,338 | 412,660 | 1,018 | 307,217 | 89 | 27,282 |
| Oct-16 | 261 | 61,783 | 1,266 | 410,247 | 935 | 298,051 | 80 | 29,054 |
| Jan-17 | 317 | 65,783 | 1,484 | 449,007 | 990 | 324,399 | 114 | 29,804 |
| Apr-17 | 275 | 72,139 | 1,704 | 466,862 | 1,178 | 325,921 | 125 | 30,082 |
| Jul-17 | 212 | 70,853 | 1,516 | 457,136 | 969 | 324,344 | 106 | 31,621 |
| Oct-17 | 261 | 71,844 | 1,468 | 459,053 | 891 | 318,689 | 111 | 31,743 |
| Jan-18 | 277 | 75,980 | 1,622 | 493,161 | 1,077 | 342,347 | 65 | 33,562 |
| Apr-18 | 264 | 79,022 | 1,569 | 512,927 | 1,236 | 346,617 | 127 | 33,167 |
| Jul-18 | 238 | 79,608 | 1,550 | 504,072 | 1,077 | 338,863 | 116 | 33,764 |
| Oct-18 | 261 | 83,378 | 1,400 | 490,176 | 924 | 314,211 | 131 | 36,092 |
| Jan-19 | 294 | 89,423 | 1,393 | 526,795 | 1,100 | 346,972 | 129 | 34,474 |

© 2025 Ahmedani BK et al. *JAMA Network Open*.

| | | | | | | | | |
|---|---|---|---|---|---|---|---|---|
| Apr-19 | 358 | 100,243 | 1,522 | 542,185 | 1,212 | 352,329 | 133 | 35,071 |
| Jul-19 | 308 | 101,487 | 1,423 | 538,932 | 1,071 | 351,898 | 140 | 33,303 |
| Oct-19 | 304 | 106,084 | 1,250 | 507,134 | 865 | 322,217 | 114 | 34,121 |

© 2025 Ahmedani BK et al. *JAMA Network Open*.
