# Supplementary material for: Zero Suicide Model Implementation and Suicide Attempt Rates in Outpatient Mental Health Care
Source: JAMA Netw Open. 2025 Apr 7;8(4):e253721. doi: 10.1001/jamanetworkopen.2025.3721 (PMC11976489; doi:10.1001/jamanetworkopen.2025.3721)
Supplement: Supplement 2. — Data Sharing Statement [file jamanetwopen-e253721-s002.pdf]

Data Sharing Statement

Ahmedani. Zero Suicide Model Implementation and Suicide Attempt Rates in Outpatient Mental Health Care. *JAMA Netw Open*. Published April 07, 2025.
doi:10.1001/jamanetworkopen.2025.3721

Data
Data available: Yes
Data types: Data dictionary
How to access data: bahmeda1@hfhs.org
When available: With publication

Supporting Documents
Document types: Statistical/analytic code
How to access documents: bahmeda1@hfhs.org
When available: With publication

Additional Information
Who can access the data: researchers whose proposed use of the data has been approved
Types of analyses: aggregate data
Mechanisms of data availability: after approval of a proposal
